# Diagnostic Challenges in Pleural Mesothelioma

**DOI:** 10.3390/cancers18091374

**Published:** 2026-04-25

**Authors:** Moshe Lapidot, Martin Sattler

**Affiliations:** 1 Division of Thoracic Surgery, Lung Center and International Mesothelioma Program, Brigham and Women’s Hospital and Harvard Medical School, Boston, MA 02115, USA; 2 Department of Thoracic Surgery, Galilee Medical Center, Nahariya 2210001, Israel; 3 Department of Medical Oncology and Therapeutics Research, City of Hope National Medical Center, Duarte, CA 91010, USA; msattler@coh.org

**Keywords:** pleural mesothelioma, diagnosis, asbestos, biomarker

## Abstract

Accurate diagnosis of PM and precise histologic subtyping are critical for optimal therapeutic decision-making, as treatment strategies—including chemotherapy, immunotherapy, or multimodality approaches—are largely subtype-dependent. Because of the several-decade latency between fiber inhalation and symptom onset, many cases are diagnosed at an advanced stage, when patients are already in poor clinical condition. As observed across multiple solid malignancies, earlier-stage diagnosis is associated with improved prognosis and expanded therapeutic options. However, the rarity of PM, the absence of validated screening strategies, and its nonspecific clinical and radiologic presentation—often mimicking both benign and metastatic pleural conditions, frequently result in diagnostic delay. Furthermore, the lack of pathognomonic histopathologic markers further complicates timely and definitive diagnosis. This review aims to delineate the epidemiologic, clinical, radiologic, and pathologic barriers that hinder accurate and early detection of PM. Current clinical evidence points to an urgent need to develop novel, validated biomarkers in PM, which will require a multidisciplinary approach.

## Simple Summary

Pleural mesothelioma (PM) is a rare and highly aggressive malignancy, with an incidence of approximately 1 per 100,000 individuals in the United States. Asbestos exposure constitutes the predominant and near-exclusive etiologic determinant. The diagnosis of PM is established by integrating clinical and radiologic findings with morphologic evaluation, immunohistochemical profiling, and, if needed, molecular analyses. The disease’s low incidence, extended latency period following exposure, nonspecific clinical presentation, and intrinsic diagnostic complexity on radiologic and histopathologic grounds collectively contribute to delayed and frequently advanced-stage diagnosis. Despite continued efforts to identify biomarkers for the early detection of PM, none have yet been validated for routine clinical use.

## 1. Introduction

PM is an uncommon and markedly aggressive malignancy, with a 5-year overall survival rate of approximately 5–10% [1]. Over the past decade, the therapeutic landscape of PM has evolved substantially. Regulatory approval of immune checkpoint inhibitors has improved overall survival, particularly among patients with non-epithelioid subtypes [2,3]. Concurrently, many centers worldwide have shifted from extrapleural pneumonectomy (EPP) to pleurectomy decortication (PD), reflecting lower perioperative morbidity and mortality and comparable—if not superior—long-term outcomes [4]. PD, within a multimodality treatment framework, is recommended by both NCCN and ASCO guidelines for carefully selected patients with early-stage disease [5,6]. In one of the largest published surgical series of PD, multivariable analysis identified early T stage and lower preoperative tumor burden—quantified by volumetric assessment—as independent prognostic factors [7]. Within this cohort, patients with epithelioid PM and T1 disease achieved a 5-year survival rate of 54.1%. Accordingly, identifying PM at an early stage may improve overall survival while expanding available therapeutic options. The search for diagnostic biomarkers has encompassed a wide range of omics domains, including proteomics, transcriptomics, epigenomics, and volatomics. Despite numerous studies and some promising findings, no diagnostic biomarker has yet been successfully translated into clinical practice. The present manuscript aims to delineate the persistent challenges in achieving early and timely diagnosis of PM despite the presence of a well-established risk factor.

## 2. Epidemiology and Screening

PM is an uncommon malignancy, with an incidence of approximately 1 per 100,000 individuals in the United States, corresponding to nearly 3300 new cases annually [8,9]. The link between asbestos exposure and PM has been well established and poses one of the strongest known associations between any cancer and a single environmental cause. The risk of developing PM increases over time with cumulative exposure [10,11]. Asbestos is banned in most Western countries, but due to its extensive historical use, the United Kingdom and Australia report some of the highest incidence rates in the Western world, with ~2.9 cases per 100,000 persons per year [12,13]. Nevertheless, the risk of developing PM after heavy exposure is relatively small (~1–10%), and it is even smaller for people with primarily environmental exposure [10,11,12]. A characteristic of PM is its long latency, with symptoms typically emerging 20–50 years after exposure. For this reason, some patients may be unclear or have forgotten prior asbestos exposure, particularly if it occurred indirectly, whereas others have well-documented exposure histories. Due to the well-established risk factor and the ability to identify at least some high-risk populations, there is considerable effort to developing early detection strategies, including: (a) imaging approaches, such as low-dose computed tomography (LDCT) (Table 1), (b) blood-based and molecular biomarkers, including mesothelin, fibulin-3, osteopontin, calretinin, microRNAs, and (c) volatile organic compounds (VOCs) [14,15,16,17]. In one screening study of 516 high-risk individuals with prior asbestos exposure at least 20 years ago or documented pleural plaques (most of the participants were also smokers), LDCT detected malignancy in 2.1% of participants (2 pleural mesothelioma, 6 lung cancers, 2 peritoneal mesothelioma) [17,18]. Exhaled breath containing VOCs was examined in a proof-of-concept study to discriminate between PM patients and at-risk subjects, with results showing high sensitivity and negative predictive value, suggesting potential usefulness for ruling out disease in at-risk populations [19]. Further independent validation of this small study would be needed for clinical adaptation. Also, mesothelin is commonly expressed by most PM tumors and can be detected in serum; however, it may only help monitor treatment response and is not recommended as a diagnostic test due to limited sensitivity [20,21]. Unfortunately, none of the potential biomarkers studied so far have demonstrated sufficient accuracy or clinical validity to support their use as routine screening markers in high-risk populations. Both low incidence and long latency of PM make effective population-level screening particularly challenging. There is consensus in the literature that, despite the apparent importance of early detection, considering the aggressive nature of PM, there is currently insufficient evidence to justify screening asymptomatic individuals with known asbestos exposure [11,14,22].

It is noteworthy that, despite the strong association between PM and asbestos exposure, lung cancer is more commonly observed among exposed individuals. In a meta-analysis of seven cohort studies involving 5074 asbestos-exposed workers, the prevalence of detected lung cancer was 1.1%. The authors concluded that the detection rate of lung cancer and stage I disease, using CT screening in asbestos-exposed workers, is at least comparable to that observed in heavy smokers [18]. Importantly, while professional bodies such as the American College of Radiology and the European Society of Thoracic Imaging recommend imaging and surveillance using LDCT for lung cancer in asbestos-exposed populations, these guidelines do not extend to routine screening for mesothelioma [23,24,25].

**Table 1:** Pleural mesothelioma (PM) detection rates reported in screening studies of asbestos-exposed individuals using Low Dose Computed Tomography (LDCT).

Article	Study Design	Type of Screening	Aim of the Study	Inclusion & Exclusion Criteria	Number of Patients	Findings	Remarks
**Fasola et al. (Atom 002) 2007** [26]	Prospective	Low Dose Computed Tomography (LDCT).	Early diagnosis of lung cancer and Pleural mesothelioma (PM) in the asbestos-exposed population.	Inclusion: Definitive exposure to asbestos, age 40–75 y Exclusion: * Prior cancer (other than nonmelanoma skin cancer) * Severe concomitant conditions. * No chest CT scan in the past 2 years.	Between 2.2002 and 10.2003, 1045 asbestos-exposed individuals and prior workers.	* Detection of nine cases of lung cancer and One thymic carcinoid case. No pleural mesothelioma was detected. * The overall detection rate was 1% of the enrolled population.	Limitations: short observation study, small group. The authors state,” The role of LDCT in screening for PM remains uncertain.”
**Roberts et al. 2009** [17]	Prospective	Low Dose Computed Tomography (LDCT).	Screening for malignant asbestos-related lung diseases.	Inclusion: * Asbestos exposure- at least 20 years ago or documented pleural plaques on CXR. * Age ≤ 80 y, general good health without signs or symptoms of pleural chest disease Exclusion: Prior cancers (except for no melanoma skin cancer)	Between 3.2005 and 10.2007 516 asbestos workers. Age 32–80 years old	* Overall detection rate—2.1%: Six Lung cancer. Two Pleural mesotheliomas. Two Peritoneal mesotheliomas. Lung cancer detection rate 1.1% Four cancers were detected after the baseline scan.	Limitations: short observation study. Small group. The authors state, “We expect to learn more about the appearance of ‘early mesothelioma’ with continued screening”.
**Barbone et al. 2018** **Follow-up study of ATOM 002.** [27].	Retrospective	Low Dose Computed Tomography (LDCT).	Assessment of mortality reduction from lung cancer in participants of the ATOM 002 trial	Based on the participation criteria in the Atom trial Exclusion: * Cancer diagnosis on enrollment. * Records that could not be linked with the regional health database * Lost to follow-up participants * Women.	Between 2002 and 2011, 926 participants of the ATOM 002 study	* Sixteen cases of lung cancer and eight cases of PM were detected. * 59% reduction in lung cancer mortality with LDCT. No mortality reduction in pleural mesothelioma	Limitation: The study was not powered to compare mortality for outcomes other than lung cancer.
**Kato et al. 2018** [28].	Prospective	Low Dose Computed Tomography (LDCT).	Prevalence of lung cancer and pleural mesothelioma in the past asbestos-exposed population	* Engagement in: (a) Asbestos product manufacturing > 1 y or (b) Other industries related to asbestos exposure > 10 y or (c) Other industries related to asbestos exposure plus pleural plaques.	Between 2010 and 2012, 2132 asbestos-exposed subjects	Forty-five cases of lung cancer (2.1%) Seven cases of pleural mesothelioma (0.3%)	The authors state, “The usefulness of LDCT for early diagnosis and treatment outcome in pleural mesothelioma should be addressed and investigated in future studies”.
**De Maria et al. 2024** [29]	Retrospective	Chest X-Ray and Low Dose Computed Tomography (LDCT).	Describing pathologies in a health surveillance program for asbestos-exposed workers.	* Self-reported asbestos exposure or * Evidence of working in a well-known sector for asbestos exposure	1994–2020, 1405 asbestos-exposed subjects. Three hundred thirty-nine (24% of the cohort) were subjected to surveillance.	Detected sixty-nine cases of pleural mesothelioma (20.3% of 339 cases) And twenty cases of lung cancer (5.8% of 339 cases)	

## 3. Clinical Presentation

The early development of PM is usually asymptomatic, and during progression, common symptoms that can overlap with other pleural diseases can appear, making early suspicion of PM involvement challenging [1,30,31]. Due to the lack of disease-specific symptoms, the diagnosis of PM is typically delayed by about three months from the onset of symptoms, or even more in regions where the disease is rare. Dyspnea often results from gradual pleural fluid accumulation, causing exertional breathlessness, or from tumor encasement of the lung, while chest pain usually reflects direct invasion of the chest wall. Although nonspecific and potentially attributable to a range of infectious, inflammatory, or malignant conditions, the presence of these symptoms—particularly in at-risk individuals—should prompt consideration of PM. Less frequent presenting features, often arising as the tumor progresses, include weight loss, fatigue, fever, night sweats, anorexia, thrombocytosis, hypoalbuminemia, anemia, and elevated erythrocyte sedimentation rate [1].

## 4. Radiological Presentation

Chest radiography is frequently used due to its low cost and widespread availability for exploring the nonspecific nature of presenting symptoms. Findings such as pleural thickening and pleural effusion are nonspecific, as inflammatory conditions and pleural metastases are considerably more common in other diseases than PM. Consequently, chest radiographs with equivocal or suspicious findings—especially in patients with calcified pleural plaques or documented asbestos exposure—often lead to further evaluation with cross-sectional imaging. However, computed tomography (CT) is primarily used for imaging of PM, allowing for the assessment of the extent of the disease, but it often cannot reliably distinguish malignant from benign pleural changes. There are also limitations in regard to the estimation of tumor infiltration or detection of early-stage disease, especially when there is no extensive pleural thickening or fluid accumulation [32,33]. The inclusion of intravenous contrast can improve CT imaging and help evaluate the involvement of the mediastinal pleura, interlobar fissures, and circumferential tumor growth along the pleural surface [34]. Even though positron emission tomography-computed tomography (PET-CT) and magnetic resonance imaging (MRI) are frequently used, neither can provide pathognomonic findings. One of the major limitations of CT is the identification of lymph node involvement or PM outside the thorax, which can be overcome by PET-CT. On the other hand, false-positive results can occur with infection or inflammation, and the signal strength does not necessarily correlate with tumor subtype or tumor burden [1,35].

Owing to its higher contrast resolution, MRI more effectively than a CT scan distinguishes a pleural tumor from benign fibrous tissue. The integration of signal characteristics with morphologic features yields greater sensitivity and specificity for detecting pleural malignancy [36]. Interestingly, in a small cohort of 31 patients, Usuda et al. demonstrated the value of diffusion-weighted magnetic resonance imaging (DWI) in distinguishing malignant from benign pleural disease and even in differentiating pleural mesothelioma from pleural metastases of lung cancer [37]. Today, MRI is known to be particularly useful for evaluating invasion of the chest wall, mediastinum, and diaphragm, but its use is constrained by limited availability, longer acquisition times, and high costs. To address the challenges in evaluating pleural thickness and differentiating malignant from benign disease, radiomics models have been employed. However, Peña et al. demonstrated that the most effective model based on CT and MRI scans did not outperform the diagnostic decisions made by thoracic radiologists [38].

From a clinical and pathological perspective, imaging findings are often nonspecific and must be integrated with histological and immunohistochemical data to make a definitive diagnosis. While the presence of diffuse or nodular pleural thickening on CT scans may indicate the likelihood of PM, particularly when the mediastinal pleura is affected, Imaging cannot differentiate between benign and malignant mesothelial proliferations or reliably identify mesothelioma in situ, which may present only as an unexplained pleural effusion without a mass [32,39].

## 5. Pathological Assessment

The World Health Organization (WHO) classifies mesothelial tumors into benign tumors as well as PM [40]. PM comprises three principal histologic subtypes, including epithelioid, sarcomatoid, and biphasic (containing both components). Each subtype is defined by distinct biological behavior and prognosis. The most common histology of PM is epithelioid, which constitutes 60–70%, while biphasic constitutes 15–30%, and sarcomatoid constitutes 5–15%. The preinvasive lesion of PM, termed mesothelioma in situ (MIS), which is characterized by a single layer of proliferating neoplastic cells, is exceedingly rare to diagnose [41].

PM is one of the most complex and challenging thoracic diagnoses for pathologists, and many may encounter only a few cases, if any. The difficulty in establishing standardized and accurate diagnosis lies in the histologic heterogeneity, overlap with benign and malignant features, and lack of unique PM markers. To address these challenges, pathologists from the International Mesothelioma Interest Group (IMIG) have provided guidelines in 2017 that are designed to address morphologic pitfalls, provide standardized immunohistochemical marker use, and incorporate molecular testing, such as for BAP1 loss and CDKN2A deletion, to define malignant growth [42]. An additional concern is the histopathological growth patterns of PM, which can be diverse and mistaken for metastatic pleural tumors of non-mesothelial origin. Ideally, a diagnostic approach would, therefore, attempt to combine clinical, radiologic, and pathologic features with immunohistochemistry and molecular analyses to yield a highly accurate diagnosis [43,44].

Pleural effusion is a very common presentation of diffuse PM (up to 90%) [45]. Although fluid cytology can be diagnostically useful for the epithelioid subtype of mesothelioma, as epithelioid tumor cells are often present in pleural fluid and may be diagnosed with the aid of ancillary studies, sarcomatoid and biphasic cells are less frequently detected in it and are less likely to be adequately sampled, making cytologic diagnosis from pleural fluid rarely feasible for these non-epithelioid subtypes (Table 2).

Furthermore, many histologic features with prognostic significance, such as nuclear grade and architectural patterns, cannot be reliably assessed in cytological specimens. Consequently, the sensitivity of pleural fluid cytology is insufficient to guide therapeutic decision-making [6].

Importantly, mesothelial proliferations can be diagnostically challenging even in small specimens, such as small tissue biopsies [46]. Therefore, the American Society of Clinical Oncology (ASCO) emphasizes that adequate tissue sampling via thoracoscopy or image-guided biopsy is essential.

### 5.1. The Thoracic Surgeon’s Role in Supporting Pathologic Diagnosis

The diagnosis of PM and determination of its histologic subtype—epithelioid, sarcomatoid, or biphasic—depend on pleural tissue biopsies. Although the diagnosis of epithelioid PM—the most prevalent subtype—may be relatively clear and non-complicated, the sarcomatoid and biphasic variants pose substantial diagnostic challenges and are often considered diagnoses of exclusion [44].

Pathologic assessment relies primarily on histologic evaluation and immunohistochemical profiling. For a definitive diagnosis of PM, thoracoscopic pleural biopsies are considered the gold standard, aiming to obtain multiple tissue samples while minimizing the number of ports used [5,47]. The European Society of Thoracic Surgeons (ESTS) strongly recommends obtaining multiple deep tissue biopsies via thoracoscopy [48], and the ASCO advises thoracoscopic biopsies for PM patients who are candidates for antineoplastic therapy [6]. Thoracoscopic biopsy helps confirm the histologic subtype and provides tissue for additional analyses, such as molecular profiling. For patients with unilateral pleural thickening, the European Society for Medical Oncology (ESMO) and the National Comprehensive Cancer Network (NCCN) recommend pleural sampling, preferably via thoracoscopy. To ensure reliable subtyping and grading, ESMO also recommends biopsies from at least three separate sites [49]. One of the challenges here is that a definitive diagnosis of biphasic mesothelioma in resection specimens (e.g., PD) requires at least 10% representation of both epithelioid and sarcomatoid components; this percentage threshold is not required in diagnostic biopsies. It is therefore not too surprising that postoperative histologic classification in some cases may result in changes when small sample numbers were acquired, resulting in discordances between preoperative and postoperative histologic classifications [50,51]. In one study involving 147 consecutive patients undergoing PD, concordance between preoperative and postoperative biphasic diagnoses was observed in only 83 patients; 60 patients (40.7%) were preoperatively classified as epithelioid, and 4 patients (2.7%) as sarcomatoid [50]. Current evidence highlights the importance of comprehensive tissue sampling to achieve accurate histologic subtyping. In our practice, we obtain at least three thoracoscopic biopsies from different areas of the parietal pleura. Each biopsy is taken deep enough to include the endothoracic fascia [52]. To mitigate the unique risk of PM recurrence along incision or drain tracts, we advocate the use of a single thoracoscopic port for diagnostic procedures. The position should be along the planned thoracotomy incision line to allow en bloc excision during PD [47], and this recommendation is in line with the updated 2025 ASCO guidelines [6].

Video-assisted thoracoscopic surgery (VATS)—guided pleural biopsy is an excellent tool that has demonstrated outstanding diagnostic performance, achieving a sensitivity of 93% and a specificity of 100% (Table 2). However, a major challenge is to distinguish PM from benign disease, such as chronic pleuritis or benign mesothelial proliferation, and to identify tumor cells that are masked by inflammation or fibrotic growth. In the rare desmoplastic subtype, tumor cells are often scarce and masked by dense fibrotic stroma; therefore, a definitive diagnosis, even with extensive tissue sampling, can be challenging [21]. Tissue yield can be limited in patients with restricted access to the pleural cavity, such as those with extensive pleural adhesions or minimal effusion, resulting in delayed diagnosis. Also, cases in which the initial VATS biopsy demonstrates nonspecific pleuritis are prone to definitive diagnostic delay, which can reach a median interval of 160 days [44]. Importantly, when clinical suspicion persists despite a negative VATS biopsy, repeat tissue sampling should be strongly considered, especially in individuals with documented asbestos exposure [53].

### 5.2. Immunohistochemistry (IHC)

The diagnosis of PM is somewhat different from other cancers, as IHC requires a differential approach. As for other cancers, the tissue of origin will be determined, but it is also necessary to distinguish between malignant and benign reactive disease of mesothelial origin. After it has been determined that the pleural specimen is of mesothelial origin, the next step is to exclude benign disease. PM is driven by tumor suppressors, and the loss of their expression further supports diagnosis. This can be done by IHC or molecular tests.

Defining mesothelial lineage: IHC is an essential step for the diagnosis of PM. Unfortunately, no single marker has been identified that is reliable and sufficient on its own. In addition, staining patterns among the three histologic PM subtypes can differ, and even within these groups, the sensitivity and specificity of the markers can vary. To overcome these limitations, diagnosis typically relies on two sets of markers: at least two that are consistent with a mesothelial origin and two that help rule out other malignancies. In particular, lung cancers have the propensity to metastasize and cause pleural disease, which needs to be excluded for proper diagnosis [45].

Common markers for mesothelial lineage cells include calretinin, cytokeratin (CK) 5/6, Wilms tumor-1 (WT1), HEG1, and podoplanin (D2-40), whereas markers used to exclude other malignancies include TTF-1, Napsin A, MOC-31, polyclonal CEA, Ber-EP4, and claudin-4. Importantly, the diagnostic performance of mesothelial lineage is very good for the epithelioid subtype but reduced in the sarcomatoid subtypes, where immunophenotypic expression may be limited. A principal diagnostic challenge for pathologists is distinguishing pleural involvement by sarcomatoid tumors, such as sarcomas and pleomorphic carcinoma of the lung, from sarcomatoid pleural mesothelioma. This challenge arises from overlapping morphologic features, reduced sensitivity of mesothelial markers, and shared cytokeratin expression in both tumor types. In general, in sarcomatoid PM, the carcinoma-associated markers such as claudin-4 are often of limited utility, and the immunohistochemical evaluation needed for this subtype histology is more comprehensive than that of the epithelioid. In this context, the lesion’s morphologic characteristics play a critical role in guiding the selection of additional immunohistochemical stains.

Malignant versus benign disease: Malignant growth is primarily distinguished from benign mesothelial proliferation through histopathological features, such as stromal invasion, tumor necrosis, and disorganized growth [45]. Determining these features can be supplemented by adjunct assays that measure loss in tumor suppressors commonly observed in PM. BAP1 loss is seen in approximately 50–70% of epithelioid mesotheliomas, but less frequently in sarcomatoid subtypes or MIS, and is rare in benign/reactive proliferations, yielding specificity near 100% but moderate sensitivity (60–69%) [54,55]. It means that a lack of BAP-1 staining in mesothelial cells is diagnostic of malignancy. 5′-methylthioadenosine phosphorylase (MTAP) gene situated on chromosome 9p21 and in proximity to the CDKN2A gene. The majority of 75–90% of PM with CDKN2A deletion demonstrate codeletion of MTAP [56]. Importantly, loss of cytoplasmic MTAP IHC is highly specific for the deletion of CDKN2A and 100% specific for PM diagnosis. Immunohistochemical detection of BRCA-associated protein 1 (BAP1), 5′-methylthioadenosine phosphorylase (MTAP), or FISH analysis for CDKN2A deletion takes advantage of these changes to enhance accuracy in diagnostically challenging cases and thereby differentiate malignant from benign mesothelial proliferations (such as cell hyperplasia and organizing pleuritis).

### 5.3. Adding Molecular Tests for Challenging Cases

CDKN2A homozygous deletion is found in nearly 70% of PM and can be detected by Fluorescence in situ Hybridization (FISH). Homozygous deletion shows 100% specificity and moderate sensitivity (59–69%) for PM and is absent in benign reactive conditions [57,58]. Homozygous deletion is more frequent in sarcomatoid PM than in the epithelioid subtype. In difficult cases where distinguishing benign from malignant lesions is challenging, and both nuclear BAP1 and cytoplasmic MTAP IHC staining are retained, FISH analysis for CDKN2A deletion becomes necessary. Loss of a single copy of NF2 (neurofibromin 2/merlin), detectable by FISH, occurs in roughly half of PM cases, placing it among the most commonly inactivated tumor suppressor genes together with BAP1 and CDKN2A. NF2 is now increasingly understood to play a central role in modulating both cellular metabolism and the immune microenvironment in pleural mesothelioma [59,60]. Assessment of hemizygous NF2 loss by FISH, which occurs more frequently in sarcomatoid PM, may aid in the diagnosis of challenging cases [61,62].

An integrative diagnostic approach based on NCCN 2026 guidelines is summarized in Table 3.

## 6. Investigational Approaches

Given the diagnostic challenges detailed above, considerable efforts have focused on identifying biomarkers for PM using omics-based liquid biopsy approaches. Seven major omics fields—genomics, epigenomics, transcriptomics, proteomics, metabolomics, volatomics, and radiomics—have been investigated. Analyses of circulating tumor DNA (ctDNA) and cell-free DNA (cfDNA) in plasma and pleural fluid have enabled the detection of somatic mutations. Notably, Connor et al. reported ctDNA detection in 75% of PM patients, with levels showing correlation with radiologic findings. However, ctDNA is currently utilized primarily for disease monitoring and surveillance rather than for diagnostic purposes [63].

The requirement for an effective biomarker is to distinguish patients with PM from a variety of control groups, including individuals without asbestos exposure, others with varying durations of asbestos exposure (short versus prolonged), and with differing radiologic findings (benign asbestos-related pleural disease versus normal pleura). Due to PM’s low incidence, a diagnostic marker requires very high sensitivity, at least 95%, to reliably rule out disease, while also achieving high specificity to minimize false positives and unnecessary invasive procedures. Importantly, the biological heterogeneity among PM histological subtypes may influence biomarker performance, leading to variations in sensitivity, specificity, and area under the curve (AUC) across different groups.

While a range of biomarkers has demonstrated diagnostic potential, none has yet been validated for use as a single, reliable marker in clinical practice. In this paragraph, we present several representative biomarkers with high sensitivity and/or specificity.

In proteomic analyses, high specificity was achieved with a wide range of sensitivity. Johnen et al. demonstrated in a study including 116 PM patients and 147 individuals with benign asbestos-related disease (BARD) that calretinin achieved 95% specificity, AUC of 0.86, and a sensitivity of 71% [64]. In the same cohort of patients, serum mesothelin showed a comparable high specificity (95%) with a slightly higher AUC of 0.89, but a lower sensitivity of 69%.

In a study exploring the diagnostic role of soluble mesothelin-related peptide (SMRP) as a circulating tumor marker in a smaller cohort of 16 PM and 193 asbestos-exposed (AE) individuals, Smolkova et al. demonstrated that serum SMRP reached 96.2% specificity and 75% sensitivity [65]. G. Aguilar-Madrid et al. reported in 75 PM patients, 133 AE, and 107 non-exposed individuals that plasma mesothelin reached very high specificity (98–99%), though sensitivity was low; it still differed by sex, being lower in males (43%) than females (58%) [66]. In this cohort, plasma calretinin achieved perfect specificity (100%) but limited sensitivity (17–26%). When these two biomarkers were combined, modestly improved sensitivity (50–53%) was achieved in both sexes.

Plasma fibulin-3 initially appeared highly promising, with reported sensitivity and specificity of 100% and an AUC of 0.99, attracting attention as a potential standalone biomarker [67]. However, a later prospective study involving 638 patients and 110 AE individuals significantly tempered this optimism, reporting a sensitivity of only 11.9% and an AUC of 0.51 [68].

In the emerging field of volatomics, small studies have produced consistently required high sensitivity results but with a different range of specificity. Zwijsen et al. [69] demonstrated in a cohort of 6 PM and 121AE individuals 100% sensitivity but specificity of only 30%, while Lamonte et al. demonstrated in 14 PM patients sensitivity of 92.9%, specificity of 100%, and an AUC of 0.97 [70].

Within epigenomics, methylation patterns in cfDNA from pleural fluid or peripheral blood were analyzed. Hypermethylation of CCR12P was evaluated in 17 PM patients compared with a control group of 167 AE individuals stratified by exposure duration. Interestingly, the effectiveness of the biomarker depends on the duration of asbestos exposure. While among those with prolonged exposure (at least 10 years), sensitivity reached 100%, specificity 83.1%, and AUC 0.953, sensitivity dropped to 52.9% in those with shorter exposure histories defined by less than 10 years [71].

Transcriptomic approaches explored various RNA species, including messenger RNA and cell-free microRNA (miRNA), across biological samples such as serum, plasma, and leukocytes.

Reduced miRNA-126 expression in a cohort of 32 PM patients yielded 97.8% specificity, 80% sensitivity, and an AUC of 0.95 [72]. However, in a different cohort, 44 PM vs. 196 AE and 50 non-exposed individuals, sensitivity and specificity were just 73% and 74%, respectively [73].

Similarly, low leukocyte expression of miRNA-103a-3p in 108 PM patients compared with 218 AE and 19 non-exposed individuals showed high specificity (95.5%) but very low sensitivity (0–4.4%) [74]. Notably, combining the low expression signature of miRNA-103a-3p with miRNA-30e-3p in plasma (23 PM vs. 19 AE individuals) markedly improved performance, achieving 95.5% sensitivity and an AUC of 0.942 [75].

Variability across those studies and others likely reflects differences in sample type (plasma, serum, leukocyte…), control group selection, percentage of histological subtypes in the PM cohort, cohort size, and detection methodologies. While several individual biomarkers demonstrate either high sensitivity or high specificity, none has yet proven adequate for clinical application on its own. Nevertheless, combined biomarker approaches—particularly involving miRNAs—show considerable promise. Integrating multiple modalities, such as proteomics, transcriptomics, and breath analysis, may ultimately enable the development of a clinically viable test with both high sensitivity and specificity.

Immune-related parameters—including tumor-infiltrating lymphocytes (TILs), patterns of macrophage polarization, and heterogeneity in immune checkpoint expression in PM—are currently regarded mainly as prognostic and predictive indicators rather than validated diagnostic modalities. Nevertheless, several studies have suggested that these features may aid in distinguishing the most aggressive PM subtype, sarcomatoid mesothelioma, from reactive pleuritis.

In a study by Salaroglio et al., a composite signature of the immune microenvironment derived from pleural fluid and biopsy specimens in a cohort of 275 patients demonstrated the capacity to differentiate PM from pleuritis, achieving 100% sensitivity and 89% specificity [76]. These results indicate that an immunosuppressive milieu—marked by enrichment of M2-polarized macrophages, regulatory T cells, and lymphocytes expressing immune checkpoint molecules—may serve as a useful adjunct for diagnosis.

Additionally, programmed death-ligand 1 (PD-L1), an important regulator of immune responses, has shown potential diagnostic value in distinguishing sarcomatoid PM from organizing pleuritis. In a small cohort of 20 patients, strong PD-L1 expression as assessed by immunohistochemistry (IHC) was significantly associated with sarcomatoid mesothelioma [77].

## 7. Conclusions

The diagnosis of PM remains challenging and requires a stepwise approach and multidisciplinary team involvement. The rarity of the disease, its long latency period following exposure, nonspecific clinical and radiologic manifestations, and its pathological complexity all contribute to the difficulty of early detection. The role of the thoracic surgeon is important in supporting pathologic diagnosis. Obtaining pleural biopsies from multiple areas within the pleural cavity may increase the likelihood of accurately identifying the histologic subtype, which has important prognostic and therapeutic implications. Given the strong association between asbestos exposure and the development of PM, certain populations—such as asbestos workers—can be clearly identified as high-risk groups. Detecting PM at an early tumor stage (T1 or T2), or even at a pre-invasive phase such as MIS, could expand therapeutic options, including multimodal approaches, and may ultimately improve overall survival. Attempts to identify reliable biomarkers for the early detection of PM have been unsuccessful so far. Several candidates, such as SMRP, miRNA signatures, and exhaled volatile organic compounds, have shown promise but still require validation in large patient cohorts. Ultimately, a coordinated approach between mesothelioma centers that have a sufficient volume of patients may be required to address this question in randomized controlled trials. There may be a meaningful lesson to be learned from the search for effective screening tools in lung cancer. Even though lung cancer is the leading cause of cancer-related death worldwide, the development of an effective screening test remained elusive for decades. Several screening approaches, including sputum cytology, chest radiography, and even several randomized trials using LDCT, have failed to demonstrate a mortality benefit. Earlier European and American randomized controlled trials using LDCT for lung cancer screening in thousands of individuals were limited by small sample sizes, insufficient statistical power, and a lack of standardized protocols. The results were inconclusive or negative when analyzed for mortality benefit [78]. In contrast, only the NLST and NELSON trials were adequately powered to assess reductions in lung cancer mortality and employed rigorous methodology and carefully defined participant selection criteria [79,80]. Today, LDCT is widely accepted and recommended as a screening modality for the early detection of lung cancer and for reducing disease-specific mortality.

The case for PM differs from that of lung cancer in several important aspects. First, PM is a rare disease, and second, available treatments are limited and generally prolong survival rather than provide a cure for this highly aggressive malignancy. Nevertheless, survival outcomes in both surgical and non-surgical cohorts are strongly associated with the administration of treatments at early disease stages [81,82,83,84].

One of the major challenges in clinical PM research is the difficulty of recruiting enough participants due to the disease’s rarity, resulting in small, underpowered studies. There is, therefore, an essential need to foster broader international collaborations in clinical research to drive innovation. Multinational, multi-institutional collaboration is particularly critical in rare diseases such as PM to validate promising biomarkers that have yet to reach clinical implementation, including circulating miRNAs, epigenetic methylation markers, volatomic signatures, and imaging-based screening approaches. It is encouraging that efforts to establish multinational trials to address these shortcomings in PM research have begun, and that solutions are beginning to emerge following discussions at the 2025 IMIG conference.

## Figures and Tables

**Table 2:** Pleural samples for diagnosis of pleural mesothelioma (PM).

Diagnostic Sample	Invasiveness	Sensitivity%	Specificity%	Limitations and Remarks
**Pleural Effusion cytology**	low	Low sensitivity 30–75	99–100	Inability to evaluate stromal invasion. Low sensitivity for sarcomatoid mesothelioma, as it rarely sheds cells into effusions. Although the epithelioid component of biphasic mesothelioma can release mesothelioma cells into effusions, cytology cannot reliably distinguish biphasic from purely epithelioid mesothelioma. Inability to differentiate mesothelioma in situ from invasive mesothelioma. Insufficient for performing architectural subtyping or grading of epithelioid mesothelioma
**Pleural biopsies**	Intermediate	93	100	The gold standard for PM and histological subtype diagnosis.

**Table 3:** Integrative diagnostic approach for pleural mesothelioma.

Step 1	Step 2	Step 3	Step 4	Step 5
Clinical presentation	Radiological assessment	Tissue acquisition	Pathology assessment	Remarks
Most common: Recurrent pleural effusion- pleural thickening Nonspecific symptoms: Progressive dyspnea, chest pain, weight loss. Asbestos exposure history should be elucidated	Contrast-enhanced Computed Tomography scan. Assess for nodular pleural thickening, involvement of the mediastinal pleura, and circumferential (“rind-like”) encasement.	Cytology assessment from pleural fluid (mainly applicable for epithelioid PM) Pleural biopsies. Gold standard: thoracoscopic biopsy using a uniport approach, with acquisition of multiple deep tissue samples.	Histopathology assessment of malignancy: Stromal invasion, tumor necrosis, disorganized growth. Histological type determination- epithelioid, biphasic, sarcomatoid. Confirmation of Mesothelial differentiation- IHC panel Mesothelial markers (at least 2): calretinin, cytokeratin 5/6, Wilms tumor 1, HEG1, D2-40. Carcinoma markers/Negative markers (at least 2): TTF1, MOE31, polyclonal CEA, Napsin, Ber-EP4, Claudin-4. Ancillary IHC and molecular exams to confirm malignancy- BAP1 loss, MTAP loss, CDKN2A	When clinical suspicion persists despite a pathological diagnosis of reactive pleuritis, additional biopsies should be pursued.

## Data Availability

No new data were created or analyzed in this study. Data sharing is not applicable to this article.
